# Supplementary material for: Morphological Differentiation, Yield, and Cutting Time of Lolium multiflorum L. under Acid Soil Conditions in Highlands
Source: Plants (Basel). 2024 Aug 21;13(16):2331. doi: 10.3390/plants13162331 (PMC11359289; doi:10.3390/plants13162331)
Supplement: Supplementary file 1 [file plants-13-02331-s001.zip › plants-3136850-supplementary.pdf]

plants

MDPI

Suplementary 1: Genomic evaluation on LM-58 genotype

Electropherogram review

The files from the sequencing were received in a format; these files were opened using the SeqTrace 0.9.0 program to review the electropherograms. A new project was created, and the files containing the sequences were loaded (*.ab1 format). The minimum confidence value of 25 was considered a good-quality peak within the parameter settings. Subsequently, the Forward and Reverse sequences **(Figure S1)** were pooled to check the definition and quality of the peaks corresponding to each nitrogenous base. Very little or no stability was observed at the ends of the sequences, i.e. sequences of low resolution **(Figure S2)**, whereas, in the central part, where the F and R sequences complement each other, more excellent stability was observed **(Figure S3).** Considering the sound quality peaks, we generated the consensus sequence and exported them in Fasta format (*.fas).

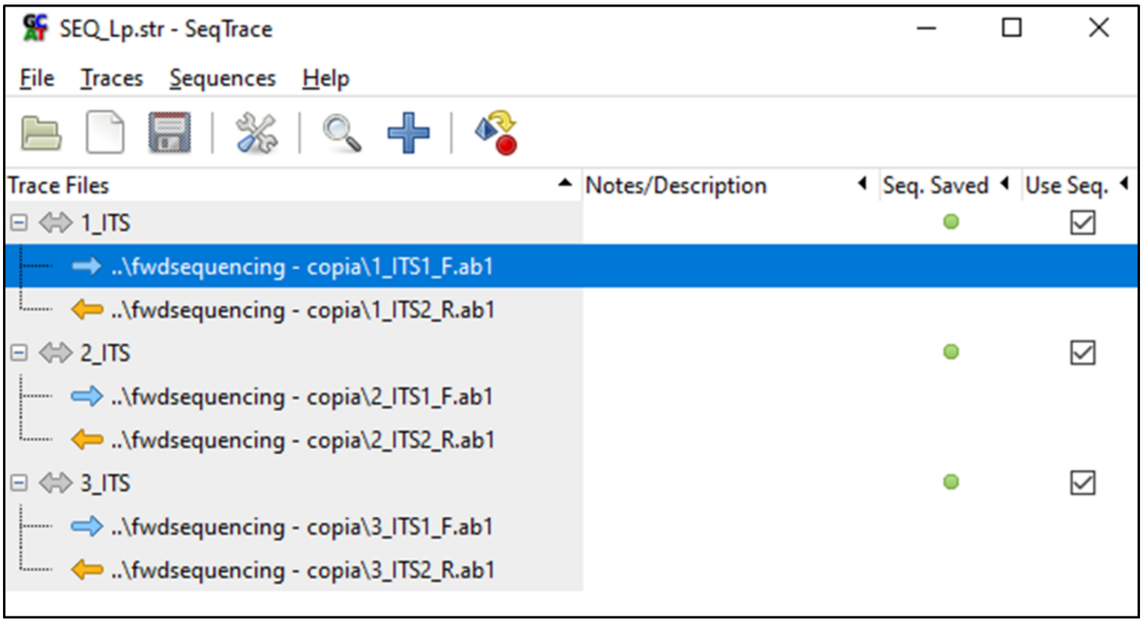

Figure S1. Grouping of Forward and Reverse sequences in the SeqTrace programme.

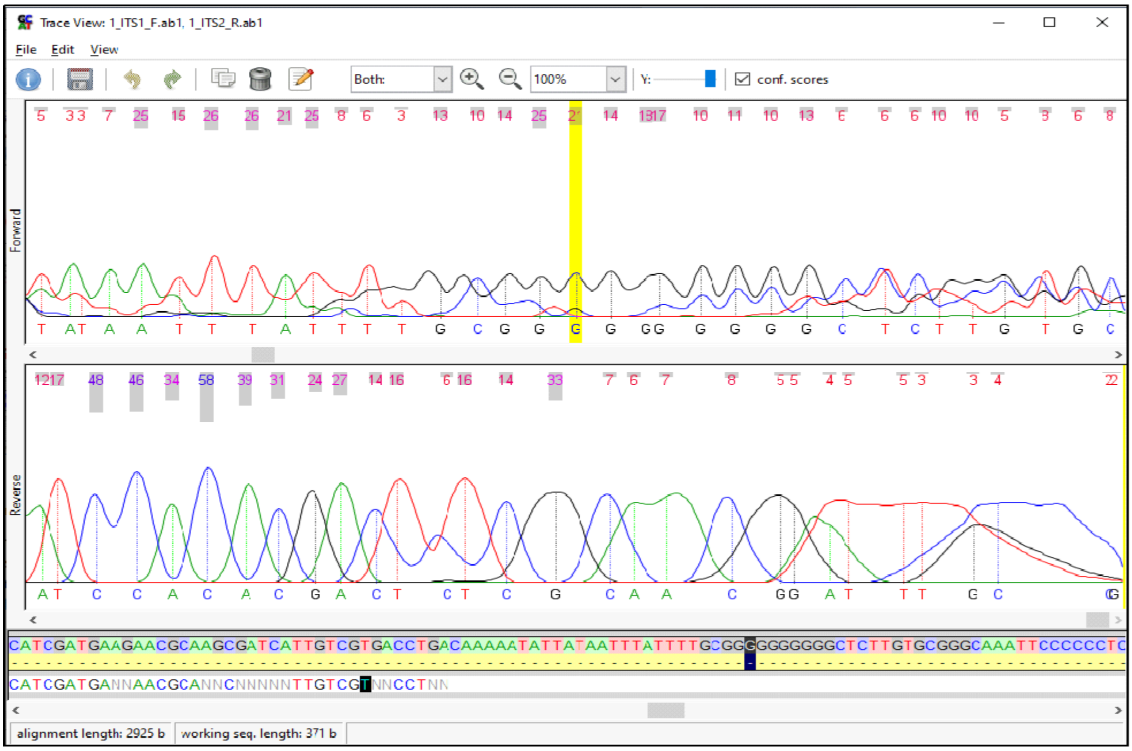

Figure S2. Poor stability of the ends of the sequences.

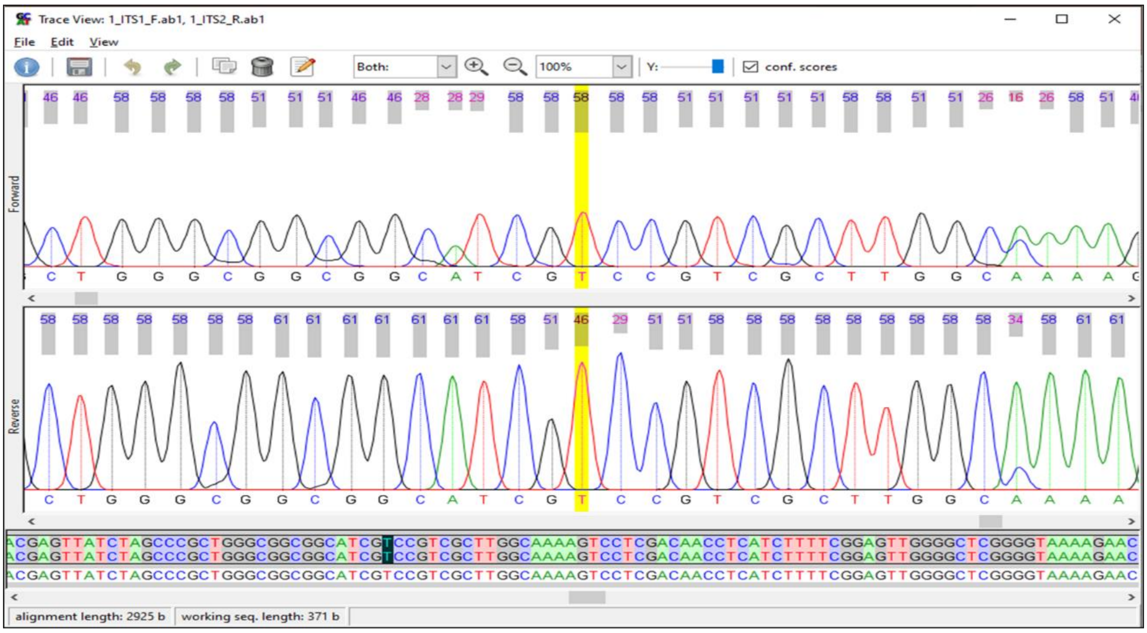

Figure S3. High stability of the central part of the sequences or complementary region.

Sequence Editing and Confirmation

Consensus sequences in Fasta format were loaded into Mega 10.1.6 and aligned using the Muscle algorithm **(Figure S4).** Subsequently, all sequences of the same size were cut (edited) and exported in Fasta (*.fas) and Mega (*.meg) format.

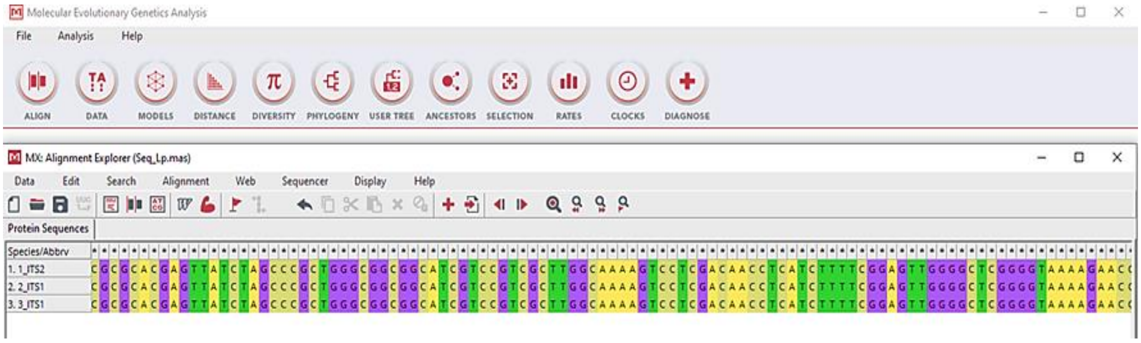

Figure S4. Sequence alignment in the Mega programme.

The generated sequences were checked against the Genebank database using the BLASTntool (https://blast.ncbi.nlm.nih.gov/Blast.cgi PAGE_TYPE=BlastSearch) to confirm the identity of the Lolium multiflorum species and also to confirm the Internal Transcribed Spacer(ITS) region of the mitochondrial DNA **(Figure S5).**

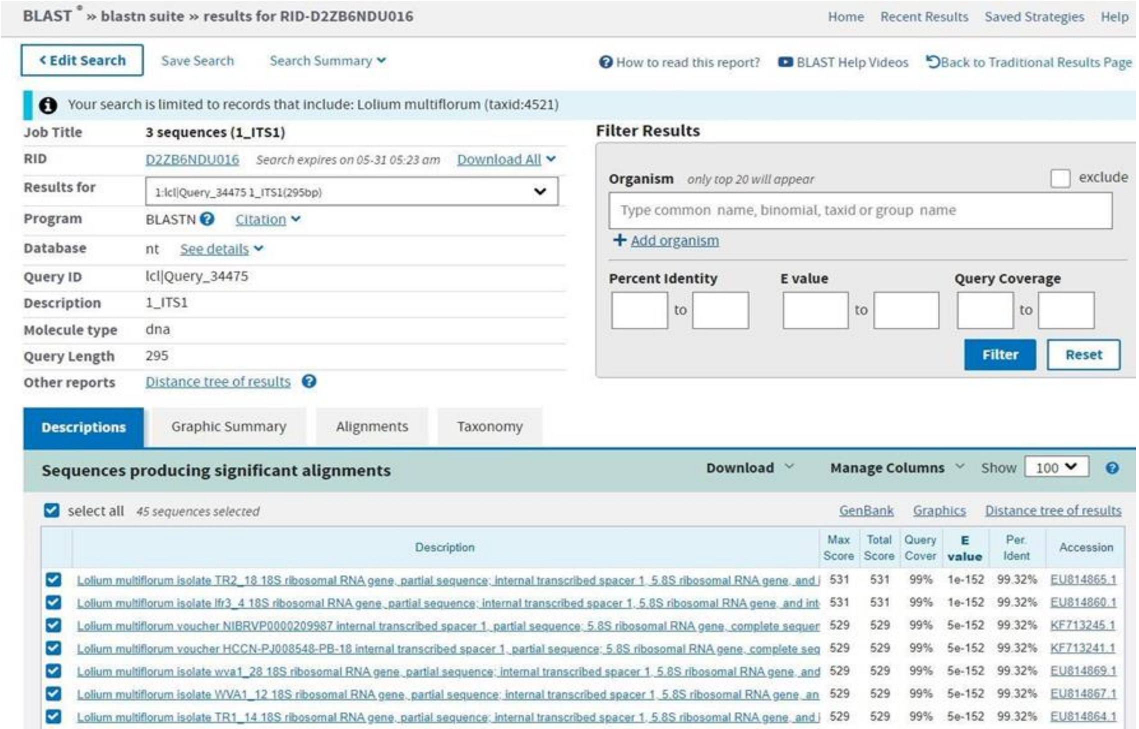

Figure S5. Identification of species and mitochondrial DNA region with the BLASTn tool.

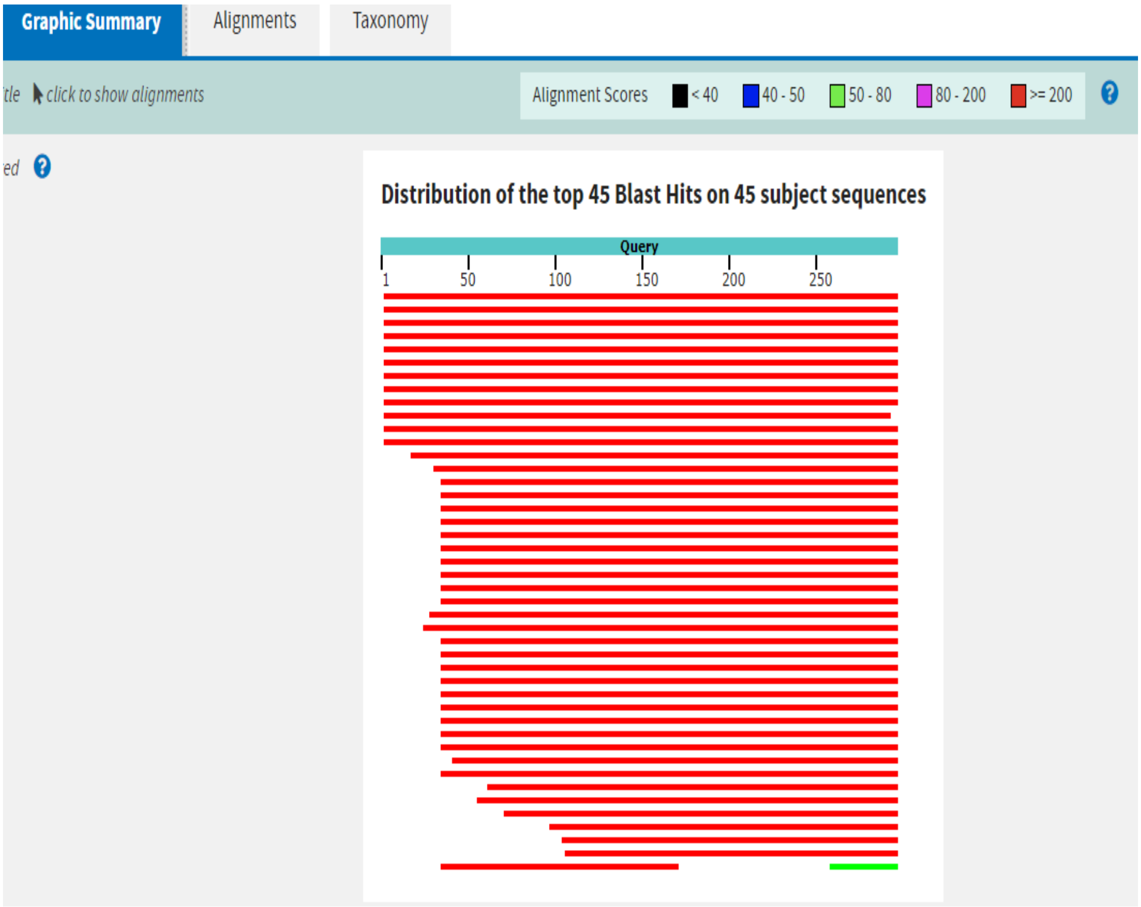

Continued Figure S5. Identification of species and mitochondrial DNA region with the BLASTn tool.

Phylogenetic trees of *Lolium multiflorum*

The phylogenetic trees of Lolium multiflorum, obtained from the mitochondrial DNA sequences, group the three samples under study and keep the reference sequence for Lolium multiflorum (Genebank) on an external branch **(Figure S6).**

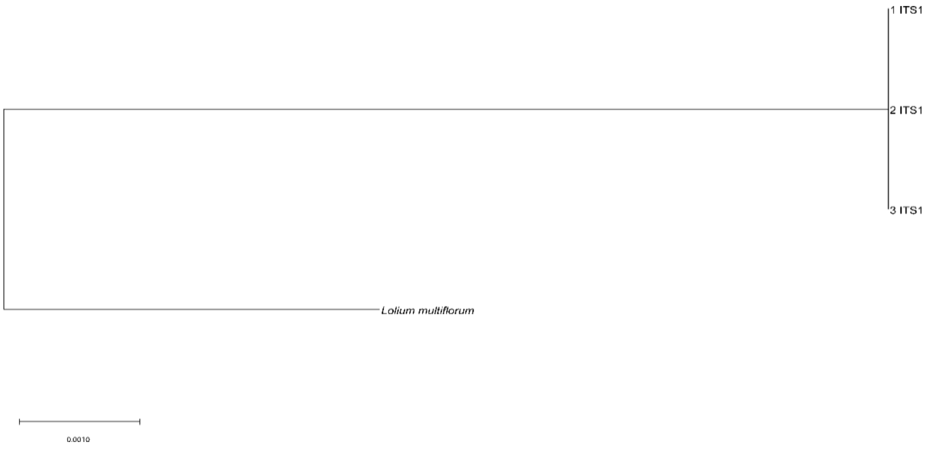

Figure S6. Neighbor-joining phylogenetic tree for *Lolium multiflorum*.

When the sequence of the Festuca drama outgroup is added, the 3 study samples maintain the clustering on a single branch. In contrast, the reference sequence and the outgroup are presented on external branches **(Figure S7).**

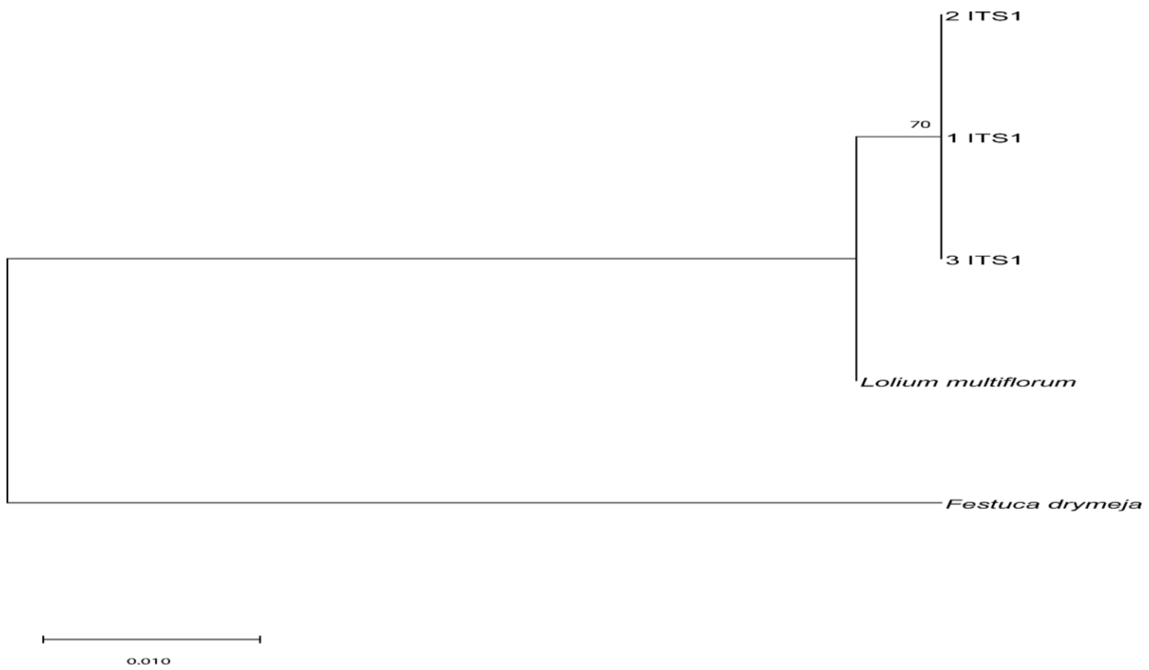

Figure S7. Phylogenetic tree Maximum Likelihood with the *Festuca drymeja* group.

Haplotype network of *Lolium multiflorum*

The haplotypic network identified for Lolium multiflorum, through the mitochondrial DNA sequences, shows two unique haplotypes, where haplotype 1 (H_1) contains the three samples under study and haplotype 2 (H_2) contains the reference sequence for *Lolium multiflorum*, obtained from Genebank **(Figure S8).**

Similarly, when the sequence of the outgroup Festuca drama is added, the 3 study samples maintain the clustering in the same haplogroup **(Figure S9).**

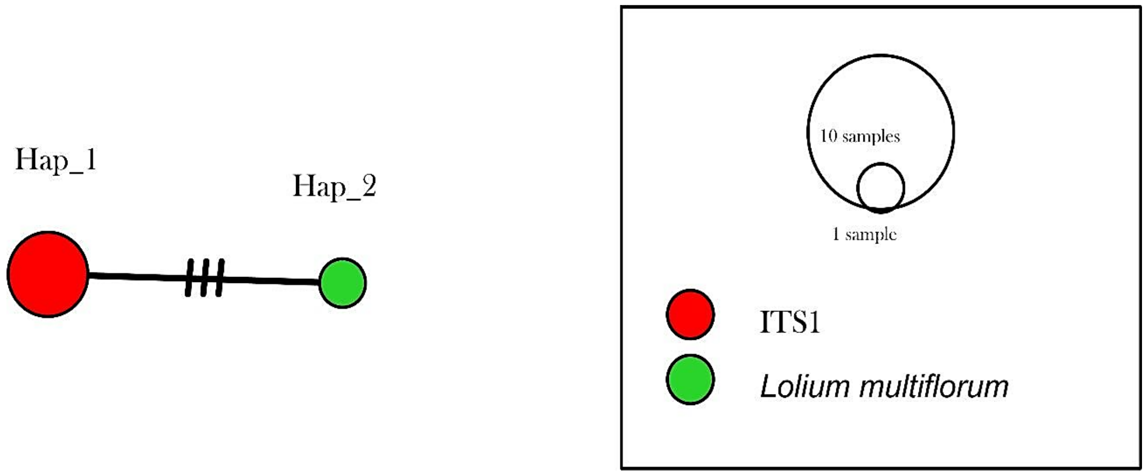

Figure S8. Haplotype network for Lolium multiflorum.

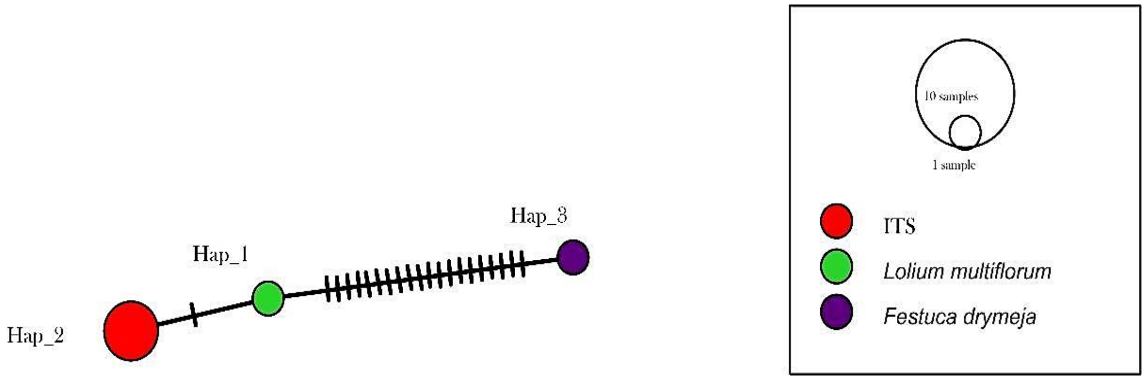

Figure S9. Haplotype network including the Festuca drymeja outgroup.
